# 2-(4-Iodo­phen­yl)-5,7-dimethyl-3-methyl­sulfinyl-1-benzofuran

**DOI:** 10.1107/S1600536808014104

**Published:** 2008-05-17

**Authors:** Hong Dae Choi, Pil Ja Seo, Byeng Wha Son, Uk Lee

**Affiliations:** a Department of Chemistry, Dongeui University, San 24 Kaya-dong, Busanjin-gu, Busan 614-714, Republic of Korea; b Department of Chemistry, Pukyong National University, 599-1 Daeyeon 3-dong, Nam-gu, Busan 608-737, Republic of Korea

## Abstract

The title compound, C_17_H_15_IO_2_S, was prepared by the oxidation of 2-(4-iodo­phen­yl)-5,7-dimethyl-3-methyl­sulfanyl-1-benzofuran using 3-chloro­peroxy­benzoic acid. The 4-iodo­phenyl ring makes a dihedral angle of 26.0 (1)° with the plane of the benzofuran fragment, and the O atom and the methyl group of the methyl­sulfinyl substituent lie on opposite sides of this plane. The crystal structure is stabilized by inter- and intra­molecular C—H⋯O hydrogen bonds, and by an I⋯O halogen bond with an I⋯O distance of 3.145 (2) Å and a nearly linear C—I⋯O angle of 164.01 (9)°.

## Related literature

For the crystal structures of similar 2-aryl-3-methyl­sulfinyl-1-benzofuran compounds, see: Choi *et al.* (2007*a* ▶,*b* ▶). For a review of halogen bonding, see: Politzer *et al.* (2007 ▶).
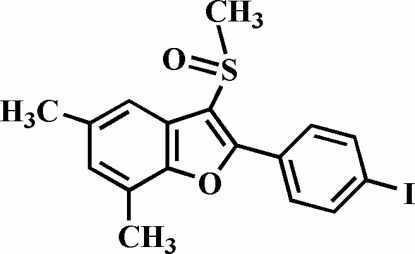


## Experimental

### 

#### Crystal data

C_17_H_15_IO_2_S
*M* *_r_* = 410.25
Triclinic, 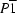

*a* = 8.6320 (9) Å
*b* = 8.917 (1) Å
*c* = 11.638 (1) Å
α = 94.580 (2)°
β = 100.949 (2)°
γ = 113.725 (2)°
*V* = 792.90 (14) Å^3^
*Z* = 2
Mo *K*α radiation
μ = 2.15 mm^−1^
*T* = 293 (2) K
0.40 × 0.20 × 0.20 mm

#### Data collection

Bruker SMART CCD diffractometer
Absorption correction: multi-scan (*SADABS*; Sheldrick, 2000 ▶) *T*_min_ = 0.594, *T*_max_ = 0.647
6882 measured reflections
3408 independent reflections
3214 reflections with *I* > 2σ(*I*)
*R*_int_ = 0.029

#### Refinement

*R*[*F* ^2^ > 2σ(*F* ^2^)] = 0.025
*wR*(*F* ^2^) = 0.080
*S* = 1.22
3408 reflections
192 parameters
H-atom parameters constrained
Δρ_max_ = 0.50 e Å^−3^
Δρ_min_ = −0.66 e Å^−3^

### 

Data collection: *SMART* (Bruker, 2001 ▶); cell refinement: *SAINT* (Bruker, 2001 ▶); data reduction: *SAINT*; program(s) used to solve structure: *SHELXS97* (Sheldrick, 2008 ▶); program(s) used to refine structure: *SHELXL97* (Sheldrick, 2008 ▶); molecular graphics: *ORTEP-3* (Farrugia, 1997 ▶) and *DIAMOND* (Brandenburg, 1998 ▶); software used to prepare material for publication: *SHELXL97*.

## Supplementary Material

Crystal structure: contains datablocks I, global. DOI: 10.1107/S1600536808014104/zl2115sup1.cif

Structure factors: contains datablocks I. DOI: 10.1107/S1600536808014104/zl2115Isup2.hkl

Additional supplementary materials:  crystallographic information; 3D view; checkCIF report

## Figures and Tables

**Table 1:** Hydrogen-bond geometry (Å, °)

*D*—H⋯*A*	*D*—H	H⋯*A*	*D*⋯*A*	*D*—H⋯*A*
C16—H16*B*⋯O1	0.96	2.55	2.975 (4)	107
C16—H16*A*⋯O2^i^	0.96	2.39	3.288 (4)	156
C17—H17*B*⋯O1^ii^	0.96	2.51	3.422 (4)	159

Symmetry codes: (i) 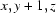; (ii) 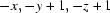.

**Fig. 1.**
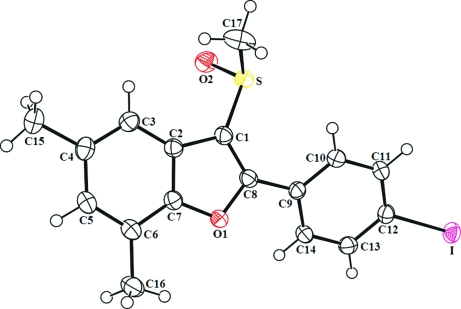
The molecular structure of the title compound, showing displacement ellipsoids drawn at the 50% probability level.

**Fig. 2.**
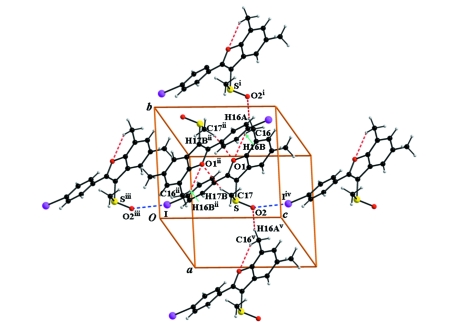
C—H···O hydrogen bond and I···O halogen bond (dotted lines) in the title compound. [Symmetry codes: (i) x, y + 1, z; (ii) -x, -y + 1, -z + 1; (iii) x, y, z - 1; (iv) x, y, z + 1; (v) x, y - 1, z.]

## supplementary crystallographic information

### Comment

As a part of our ongoing studies on the synthesis and structure of 2-aryl-3-methylsulfinyl-1-benzofuran analogues, the crystal structure of 2-(4-bromophenyl)-5-methyl-3-methylsulfinyl-1-benzofuran (Choi *et al.*, 2007*a*) and 2-(4-bromophenyl)-5,7-dimethyl-3-methylsulfinyl-1-benzofuran (Choi *et al.*, 2007*b*) have been described in the literature. Here we report the crystal structure of the title compound, 2-(4-iodophenyl)-5,7-dimethyl-3-methylsulfinyl-1-benzofuran (Fig. 1).

The benzofuran unit is essentially planar, with a mean deviation of 0.01 Å from the least-squares plane defined by the nine constituent atoms. The molecular packing (Fig. 2) is stabilized by three different C—H···O hydrogen bonds; one between a methyl H atom and the furan O atom, *i.e.* C16—H16B···O1, and a second between a methyl H atom and the oxygen of a neighbouring S═O unit, *i.e.* C16—H16A···O2^i^, and a third between a methyl H atom of the methylsulfinyl substituent and the furan O atom of neighbouring molecules, *i.e.* C17—H17B···O1^ii^, (Fig. 2 and Table 1; symmetry code as in Fig. 2). Further stabilization of the structure comes from a weak I···O halogen bond (Fig. 2) (Politzer *et al.*, 2007) between the iodine atom and the oxygen of a neighbouring S═O unit, with an I···O2^iii^ distance of 3.145 (2) Å (Symmetry code as in Fig. 2).

### Experimental

77% 3-chloroperoxybenzoic acid (359 mg, 1.60 mmol) was added in small portions to a stirred solution of 2-(4-iodophenyl)-5,7-dimethyl-3-methylsulfanyl-1-benzofuran (591 mg, 1.50 mmol) in dichloromethane (30 ml) at 273 K. After being stirred at room temperature for 2 h, the mixture was washed with saturated sodium bicarbonate solution and the organic layer was separated, dried over magnesium sulfate, filtered and concentrated in vacuum. The residue was purified by column chromatography (ethyl acetate) to afford the title compound as a colorless solid [yield 80%, m.p. 450–451 K; *R*_f_ = 0.57 (ethyl acetate)]. Single crystals suitable for X-ray diffraction were prepared by slow evaporation of a solution of the title compound in tetrahydrofuran at room temperature. Spectroscopic analysis: ^1^H NMR (CDCl_3_, 400 MHz) δ 2.44 (s, 3H), 2.53 (s, 3H), 3.10 (s, 3H), 7.03 (s, 1H), 7.59 (d, J = 8.44 Hz, 2H), 7.80 (s, 1H), 7.84 (d, J = 8.44 Hz, 2H); EI—MS 410 [*M*^+^].

### Refinement

All H atoms were geometrically located in ideal positions and refined using a riding model, with C—H = 0.95 Å for aromatic H atoms and 0.98 Å for methyl H atoms, and with *U*_iso_(H) = 1.2Ueq(C) for aromatic H atoms, and 1.5Ueq(C) for methyl H atoms.

### Crystal data

**Table:**

C_17_H_15_IO_2_S	*Z* = 2
*M**_r_* = 410.25	*F*_000_ = 404
Triclinic, *P*1	*D*_x_ = 1.718 Mg m^−^^3^
Hall symbol: -p_1	Melting point = 450–451 K
*a* = 8.6320 (9) Å	Mo *K*α radiation λ = 0.71069 Å
*b* = 8.917 (1) Å	Cell parameters from 5631 reflections
*c* = 11.638 (1) Å	θ = 2.5–28.3º
α = 94.580 (2)º	µ = 2.15 mm^−^^1^
β = 100.949 (2)º	*T* = 293 (2) K
γ = 113.725 (2)º	Block, colorless
*V* = 792.90 (14) Å^3^	0.40 × 0.20 × 0.20 mm

### Data collection

**Table:**

Bruker SMART CCD diffractometer	3408 independent reflections
Radiation source: fine-focus sealed tube	3214 reflections with *I* > 2σ(*I*)
Monochromator: graphite	*R*_int_ = 0.029
Detector resolution: 10.0 pixels mm^-1^	θ_max_ = 27.0º
*T* = 293(2) K	θ_min_ = 1.8º
φ and ω scans	*h* = −11→10
Absorption correction: multi-scan(SADABS; Sheldrick, 2000)	*k* = −11→11
*T*_min_ = 0.594, *T*_max_ = 0.647	*l* = −14→14
6882 measured reflections	

### Refinement

**Table:**

Refinement on *F*^2^	Secondary atom site location: difference Fourier map
Least-squares matrix: full	Hydrogen site location: inferred from neighbouring sites
*R*[*F*^2^ > 2σ(*F*^2^)] = 0.025	H-atom parameters constrained
*wR*(*F*^2^) = 0.080	*w* = 1/[σ^2^(*F*_o_^2^) + (0.0374*P*)^2^ + 0.4132*P*] where *P* = (*F*_o_^2^ + 2*F*_c_^2^)/3
*S* = 1.22	(Δ/σ)_max_ = 0.001
3408 reflections	Δρ_max_ = 0.50 e Å^−^^3^
192 parameters	Δρ_min_ = −0.66 e Å^−^^3^
Primary atom site location: structure-invariant direct methods	Extinction correction: none

### Special details

**Table:**

Geometry. All e.s.d.'s (except the e.s.d. in the dihedral angle between two l.s. planes) are estimated using the full covariance matrix. The cell e.s.d.'s are taken into account individually in the estimation of e.s.d.'s in distances, angles and torsion angles; correlations between e.s.d.'s in cell parameters are only used when they are defined by crystal symmetry. An approximate (isotropic) treatment of cell e.s.d.'s is used for estimating e.s.d.'s involving l.s. planes.
Refinement. Refinement of *F*^2^ against ALL reflections. The weighted *R*-factor *wR* and goodness of fit *S* are based on *F*^2^, conventional *R*-factors *R* are based on *F*, with *F* set to zero for negative *F*^2^. The threshold expression of *F*^2^ > σ(*F*^2^) is used only for calculating *R*-factors(gt) *etc*. and is not relevant to the choice of reflections for refinement. *R*-factors based on *F*^2^ are statistically about twice as large as those based on *F*, and *R*- factors based on ALL data will be even larger.

### Fractional atomic coordinates and isotropic or equivalent isotropic displacement parameters (Å^2^)

**Table:**

	*x*	*y*	*z*	*U*_iso_*/*U*_eq_	
I	0.26443 (2)	0.23987 (2)	−0.006964 (16)	0.03233 (9)	
S	0.10120 (10)	0.19886 (8)	0.60394 (7)	0.02931 (16)	
O1	0.3173 (3)	0.6586 (2)	0.54519 (18)	0.0262 (4)	
O2	0.2055 (3)	0.1813 (3)	0.7150 (2)	0.0374 (5)	
C1	0.1717 (4)	0.4134 (3)	0.6002 (3)	0.0262 (6)	
C2	0.2089 (4)	0.5443 (3)	0.6979 (3)	0.0259 (5)	
C3	0.1802 (4)	0.5533 (4)	0.8114 (3)	0.0294 (6)	
H3	0.1197	0.4569	0.8393	0.035*	
C4	0.2430 (4)	0.7078 (4)	0.8822 (3)	0.0309 (6)	
C5	0.3335 (4)	0.8521 (4)	0.8378 (3)	0.0307 (6)	
H5	0.3738	0.9548	0.8862	0.037*	
C6	0.3655 (4)	0.8489 (4)	0.7256 (3)	0.0282 (6)	
C7	0.2995 (4)	0.6914 (3)	0.6586 (3)	0.0256 (6)	
C8	0.2386 (4)	0.4876 (3)	0.5117 (3)	0.0256 (5)	
C9	0.2460 (4)	0.4269 (3)	0.3936 (2)	0.0247 (5)	
C10	0.1189 (4)	0.2744 (4)	0.3281 (3)	0.0286 (6)	
H10	0.0280	0.2098	0.3599	0.034*	
C11	0.1279 (4)	0.2191 (4)	0.2158 (3)	0.0292 (6)	
H11	0.0438	0.1169	0.1728	0.035*	
C12	0.2623 (4)	0.3162 (4)	0.1674 (3)	0.0268 (6)	
C13	0.3902 (4)	0.4699 (4)	0.2319 (3)	0.0289 (6)	
H13	0.4803	0.5348	0.1997	0.035*	
C14	0.3811 (4)	0.5239 (3)	0.3437 (3)	0.0274 (6)	
H14	0.4656	0.6259	0.3866	0.033*	
C15	0.2162 (5)	0.7226 (5)	1.0062 (3)	0.0416 (8)	
H15A	0.0937	0.6706	1.0029	0.062*	
H15B	0.2633	0.8380	1.0409	0.062*	
H15C	0.2745	0.6687	1.0538	0.062*	
C16	0.4651 (4)	1.0036 (4)	0.6797 (3)	0.0387 (7)	
H16A	0.3979	1.0671	0.6682	0.058*	
H16B	0.4867	0.9730	0.6054	0.058*	
H16C	0.5742	1.0693	0.7362	0.058*	
C17	−0.1093 (4)	0.1531 (4)	0.6283 (4)	0.0471 (9)	
H17A	−0.1675	0.0368	0.6314	0.071*	
H17B	−0.1764	0.1801	0.5646	0.071*	
H17C	−0.0976	0.2176	0.7022	0.071*	

### Atomic displacement parameters (Å^2^)

**Table:**

	*U*^11^	*U*^22^	*U*^33^	*U*^12^	*U*^13^	*U*^23^
I	0.03823 (13)	0.03425 (13)	0.02494 (12)	0.01616 (9)	0.00863 (8)	0.00135 (8)
S	0.0364 (4)	0.0219 (3)	0.0299 (4)	0.0120 (3)	0.0091 (3)	0.0058 (3)
O1	0.0313 (10)	0.0209 (9)	0.0256 (10)	0.0103 (8)	0.0077 (8)	0.0028 (8)
O2	0.0405 (12)	0.0360 (12)	0.0384 (13)	0.0193 (10)	0.0058 (10)	0.0135 (10)
C1	0.0298 (13)	0.0227 (13)	0.0263 (14)	0.0123 (11)	0.0052 (11)	0.0037 (11)
C2	0.0287 (13)	0.0234 (13)	0.0264 (14)	0.0126 (11)	0.0052 (11)	0.0045 (11)
C3	0.0312 (14)	0.0304 (14)	0.0289 (15)	0.0145 (12)	0.0085 (12)	0.0075 (12)
C4	0.0295 (14)	0.0385 (16)	0.0265 (15)	0.0166 (13)	0.0069 (11)	0.0027 (12)
C5	0.0298 (14)	0.0269 (14)	0.0318 (15)	0.0109 (12)	0.0050 (12)	−0.0023 (12)
C6	0.0240 (13)	0.0253 (13)	0.0339 (15)	0.0100 (11)	0.0068 (11)	0.0011 (11)
C7	0.0254 (13)	0.0250 (13)	0.0264 (14)	0.0117 (11)	0.0049 (11)	0.0031 (11)
C8	0.0260 (13)	0.0219 (13)	0.0269 (14)	0.0097 (10)	0.0032 (11)	0.0036 (11)
C9	0.0271 (13)	0.0237 (13)	0.0224 (13)	0.0114 (11)	0.0031 (10)	0.0042 (10)
C10	0.0275 (13)	0.0271 (14)	0.0272 (14)	0.0077 (11)	0.0060 (11)	0.0051 (11)
C11	0.0294 (14)	0.0248 (13)	0.0257 (14)	0.0067 (11)	0.0017 (11)	0.0000 (11)
C12	0.0299 (14)	0.0283 (14)	0.0233 (13)	0.0150 (11)	0.0038 (11)	0.0031 (11)
C13	0.0293 (14)	0.0277 (14)	0.0296 (15)	0.0109 (11)	0.0091 (12)	0.0069 (11)
C14	0.0276 (13)	0.0227 (13)	0.0271 (14)	0.0078 (11)	0.0037 (11)	0.0017 (11)
C15	0.0498 (19)	0.0454 (18)	0.0279 (17)	0.0184 (15)	0.0118 (15)	0.0014 (14)
C16	0.0398 (17)	0.0259 (15)	0.0469 (19)	0.0079 (13)	0.0182 (15)	0.0024 (14)
C17	0.0318 (16)	0.0336 (17)	0.076 (3)	0.0111 (14)	0.0151 (17)	0.0201 (18)

### Geometric parameters (Å, °)

**Table:**

I—C12	2.094 (3)	C9—C10	1.396 (4)
I—O2^i^	3.145 (2)	C9—C14	1.404 (4)
S—O2	1.486 (2)	C10—C11	1.387 (4)
S—C1	1.766 (3)	C10—H10	0.9300
S—C17	1.780 (4)	C11—C12	1.390 (4)
O1—C7	1.379 (3)	C11—H11	0.9300
O1—C8	1.382 (3)	C12—C13	1.401 (4)
C1—C8	1.364 (4)	C13—C14	1.378 (4)
C1—C2	1.452 (4)	C13—H13	0.9300
C2—C3	1.391 (4)	C14—H14	0.9300
C2—C7	1.396 (4)	C15—H15A	0.9600
C3—C4	1.385 (4)	C15—H15B	0.9600
C3—H3	0.9300	C15—H15C	0.9600
C4—C5	1.409 (4)	C16—H16A	0.9600
C4—C15	1.508 (4)	C16—H16B	0.9600
C5—C6	1.385 (4)	C16—H16C	0.9600
C5—H5	0.9300	C17—H17A	0.9600
C6—C7	1.385 (4)	C17—H17B	0.9600
C6—C16	1.505 (4)	C17—H17C	0.9600
C8—C9	1.459 (4)		
			
C12—I—O2^i^	164.01 (9)	C11—C10—H10	119.9
O2—S—C1	107.81 (13)	C9—C10—H10	119.9
O2—S—C17	105.98 (17)	C10—C11—C12	120.1 (3)
C1—S—C17	99.04 (15)	C10—C11—H11	119.9
C7—O1—C8	106.5 (2)	C12—C11—H11	119.9
C8—C1—C2	107.5 (2)	C11—C12—C13	120.3 (3)
C8—C1—S	123.8 (2)	C11—C12—I	119.8 (2)
C2—C1—S	127.1 (2)	C13—C12—I	119.8 (2)
C3—C2—C7	119.0 (3)	C14—C13—C12	119.4 (3)
C3—C2—C1	136.5 (3)	C14—C13—H13	120.3
C7—C2—C1	104.5 (2)	C12—C13—H13	120.3
C4—C3—C2	119.1 (3)	C13—C14—C9	120.9 (3)
C4—C3—H3	120.5	C13—C14—H14	119.5
C2—C3—H3	120.5	C9—C14—H14	119.5
C3—C4—C5	119.4 (3)	C4—C15—H15A	109.5
C3—C4—C15	120.7 (3)	C4—C15—H15B	109.5
C5—C4—C15	119.9 (3)	H15A—C15—H15B	109.5
C6—C5—C4	123.4 (3)	C4—C15—H15C	109.5
C6—C5—H5	118.3	H15A—C15—H15C	109.5
C4—C5—H5	118.3	H15B—C15—H15C	109.5
C7—C6—C5	114.7 (3)	C6—C16—H16A	109.5
C7—C6—C16	122.2 (3)	C6—C16—H16B	109.5
C5—C6—C16	123.0 (3)	H16A—C16—H16B	109.5
O1—C7—C6	124.7 (3)	C6—C16—H16C	109.5
O1—C7—C2	110.9 (2)	H16A—C16—H16C	109.5
C6—C7—C2	124.4 (3)	H16B—C16—H16C	109.5
C1—C8—O1	110.5 (2)	S—C17—H17A	109.5
C1—C8—C9	134.5 (3)	S—C17—H17B	109.5
O1—C8—C9	115.0 (2)	H17A—C17—H17B	109.5
C10—C9—C14	119.2 (3)	S—C17—H17C	109.5
C10—C9—C8	121.0 (3)	H17A—C17—H17C	109.5
C14—C9—C8	119.8 (2)	H17B—C17—H17C	109.5
C11—C10—C9	120.1 (3)		
			
O2—S—C1—C8	120.8 (3)	C1—C2—C7—O1	0.7 (3)
C17—S—C1—C8	−129.1 (3)	C3—C2—C7—C6	0.5 (4)
O2—S—C1—C2	−43.3 (3)	C1—C2—C7—C6	−178.1 (3)
C17—S—C1—C2	66.8 (3)	C2—C1—C8—O1	0.1 (3)
C8—C1—C2—C3	−178.7 (3)	S—C1—C8—O1	−166.67 (19)
S—C1—C2—C3	−12.5 (5)	C2—C1—C8—C9	179.4 (3)
C8—C1—C2—C7	−0.5 (3)	S—C1—C8—C9	12.6 (5)
S—C1—C2—C7	165.7 (2)	C7—O1—C8—C1	0.4 (3)
C7—C2—C3—C4	−0.3 (4)	C7—O1—C8—C9	−179.1 (2)
C1—C2—C3—C4	177.8 (3)	C1—C8—C9—C10	27.6 (5)
C2—C3—C4—C5	0.3 (4)	O1—C8—C9—C10	−153.1 (3)
C2—C3—C4—C15	−179.4 (3)	C1—C8—C9—C14	−153.4 (3)
C3—C4—C5—C6	−0.6 (5)	O1—C8—C9—C14	25.9 (4)
C15—C4—C5—C6	179.1 (3)	C14—C9—C10—C11	0.8 (4)
C4—C5—C6—C7	0.7 (4)	C8—C9—C10—C11	179.8 (3)
C4—C5—C6—C16	−178.9 (3)	C9—C10—C11—C12	−0.8 (4)
C8—O1—C7—C6	178.1 (3)	C10—C11—C12—C13	0.4 (4)
C8—O1—C7—C2	−0.7 (3)	C10—C11—C12—I	−175.5 (2)
C5—C6—C7—O1	−179.4 (3)	C11—C12—C13—C14	−0.1 (4)
C16—C6—C7—O1	0.2 (5)	I—C12—C13—C14	175.8 (2)
C5—C6—C7—C2	−0.7 (4)	C12—C13—C14—C9	0.2 (4)
C16—C6—C7—C2	178.9 (3)	C10—C9—C14—C13	−0.5 (4)
C3—C2—C7—O1	179.3 (2)	C8—C9—C14—C13	−179.5 (3)

Symmetry codes: (i) *x*, *y*, *z*−1.

### Hydrogen-bond geometry (Å, °)

**Table:**

*D*—H···*A*	*D*—H	H···*A*	*D*···*A*	*D*—H···*A*
C16—H16B···O1	0.96	2.55	2.975 (4)	107
C16—H16A···O2^ii^	0.96	2.39	3.288 (4)	156
C17—H17B···O1^iii^	0.96	2.51	3.422 (4)	159

Symmetry codes: (ii) *x*, *y*+1, *z*; (iii) −*x*, −*y*+1, −*z*+1.
